# Imaging and Diagnostic Tools for Cetacean Mammary Gland Assessment: Challenges and Future Directions for Marine Mammal Pathology, Medicine and Research

**DOI:** 10.1007/s10911-025-09591-7

**Published:** 2025-12-19

**Authors:** María José Robles-Malagamba, Tommaso Gerussi, Guillermo J. Sánchez-Contreras, Mason N. Dean

**Affiliations:** 1 Department of Infectious Diseases and Public Health, Jockey Club College of Veterinary Medicine and Life Sciences, City University of Hong Kong, 83 Tat Chee Avenue, Kowloon, Hong Kong, China; 2 Centre for Applied One Health Research and Policy Advice, City University of Hong Kong, 83 Tat Chee Avenue, Kowloon, Hong Kong, China; 3 Department of Comparative Biomedicine and Food Sciences (BCA), University of Padova, Legnaro, Italy; 4 The Dolphin Company, Banco Chinchorro 87, Cancún, Q.R 77504 Mexico; 5 Centre for Nature-Inspired Engineering, City University of Hong Kong, 83 Tat Chee Avenue, Kowloon, Hong Kong, China; 6 Department of Biomaterials, Max Planck Institute of Colloids & Interfaces, Am Muehlenberg 1, Potsdam, 14484 Germany

**Keywords:** Veterinary medicine, Medical imaging, Dolphin, Reproductive anatomy, Ultrasonography, Computed tomography

## Abstract

Reproduction studies are important for the conservation of cetaceans (whales, dolphins, and porpoises) because they provide essential information for assessing populations and species dynamics, particularly in relation to the management of the diverse cetacean species in human care. A vast majority of literature on the female cetacean reproductive anatomy and physiology has focused on the ovaries, which can be used to infer reproductive history, or genital diseases and anomalies. However, literature regarding the morphology, physiology, and developmental pattern of cetacean mammary glands is scarce, despite their fundamental role in providing vital nutrients for the growth of offspring. This review describes current diagnostic tools applied in human and veterinary medicine to assess mammary glands and how marine mammal medicine could benefit from incorporating these tools into standard evaluation of the mammary glands in free-ranging and captive cetaceans. By evaluating the strengths and weaknesses of the current tools used to assess the mammary glands in humans and domestic animals —such as mammography, CT, MRI and ultrasonography— we frame a collection of diagnostic approaches that might be adapted to the particular challenges faced by marine mammal veterinarians, to enhance the evaluation of cetacean mammary gland morphology, physiology and development.

## Introduction

Mammals can be differentiated from other vertebrates by their ability to produce and secrete milk from their mammary glands to feed and provide vital nutrients for the growth of their offspring [1]. This function of mammary glands is overall the same in different mammalian species. However, the development, shape, and structure of these organs can considerably vary according to reproductive and ecological adaptations [2–5]. In particular, the literature regarding the morphology and physiology of mammary glands in cetaceans (whales, dolphins and porpoises) is limited, with little investigation of inter- or intra-individual variation (e.g. according to life stage) [6, 7]. Surprisingly few anatomical and histological descriptions of mammary glands exist (e.g. in baleen whales; [8, 9]) and, only recently, more modern approaches have been used for mammary gland characterization (e.g. ultrasound examination in finless porpoises and Atlantic bottlenose dolphins; see below) [7, 10]. A recent review underlined the value of modern imaging techniques in the study of mammary gland biology and disease diagnosis, but focused only on farm and companion animals, overlooking the relevance also for zoo and aquatic animal species [11].

To our knowledge, literature on marine mammal medicine still lacks considerable baseline data (e.g. accurate ultrasonographic or volumetric descriptions of the mammary glands of small cetaceans). This review presents a brief overview of known aspects of the anatomy of the mammary glands in marine mammals, focusing on cetaceans, followed by current diagnostic tools and imaging techniques for evaluating mammary gland morphology. In discussing how diagnostic techniques have been leveraged in human and veterinary medicine, we also highlight how diverse imaging tools could be effectively incorporated in the routine examination of mammary glands in small cetaceans, taking into consideration the challenges of imaging aquatic species. By providing a literature synopsis and outlining valuable use-cases, we frame the state-of-the-art and argue for the development of standardized imaging protocols for the evaluation of cetacean mammary glands. Application of these tools to open questions in cetacean reproductive biology (e.g. physiological changes relating to demographic parameters and reproductive stages) will offer new approaches for optimizing health assessments, for populations in human care, but also for free-ranging animals.

## Anatomy and Histology of the Mammary Gland

**Fig. 1:**
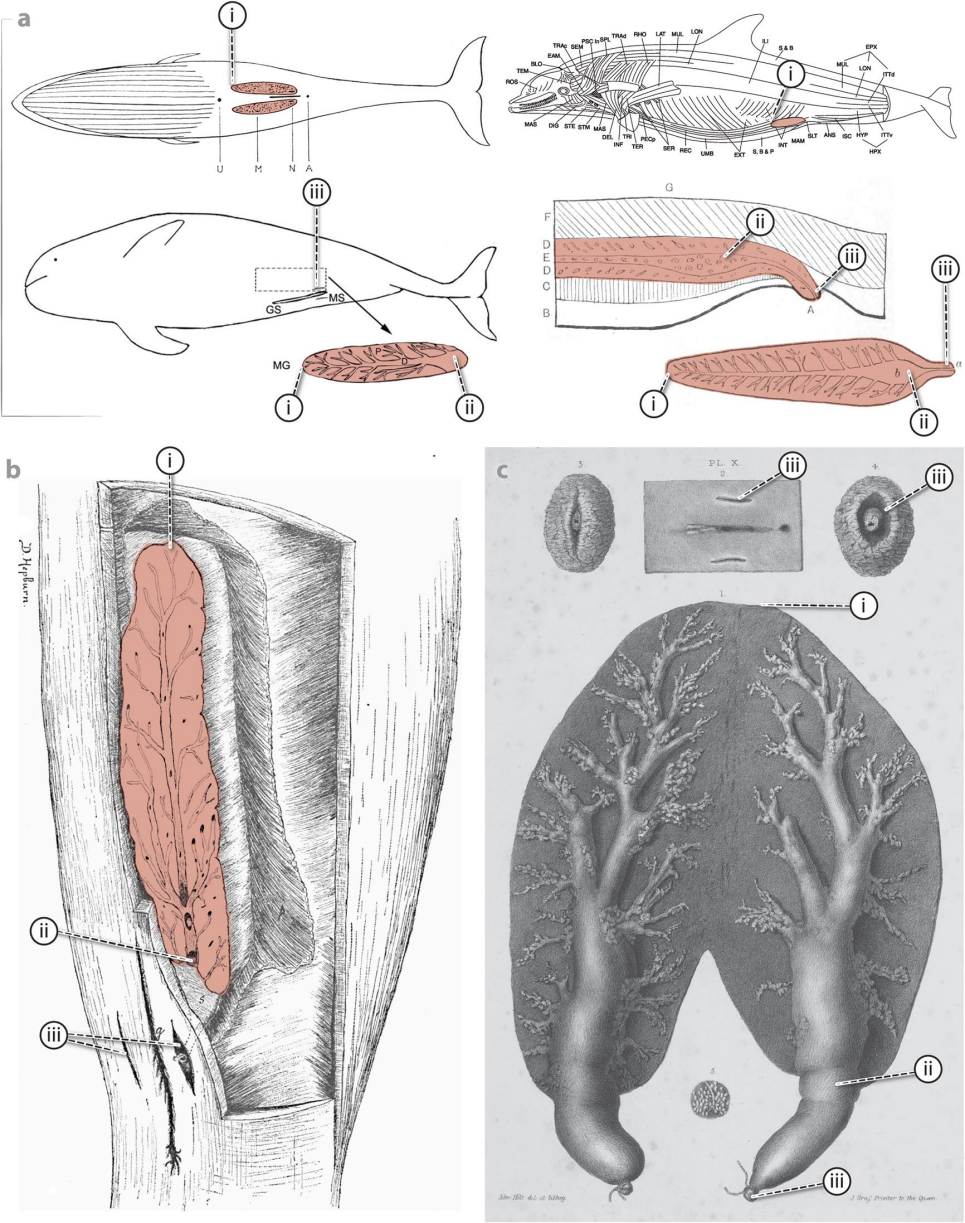
Available anatomical depictions of mammary glands (MGs) of different cetacean species, the earliest from 1840; MGs colored coral pink in all images except panel **c**. Lowercase Roman numerals: i = rostral tip of MG, ii = caudal tip/lactiferous sinus, iii = mammary slit/nipple **a** Compilation of illustrations showing the anatomical location of the glands in several species: (top left) a rorqual whale (*Balaenopteridae*) in ventral position [13]; (bottom left) a finless porpoise (*N. phocaenoides*) in right lateral recumbency showing the location of the left gland with a dashed-line rectangle, and below, the coronal section of the gland illustrating the main branch of the mammary ducts and the parenchyma [7] (reproduced with permission of John Wiley and Sons (Licence number: 6135070144572)); (top right) left lateral view of a bottlenose dolphin (*T. truncatus*) with MG depicted and superficial skeletal muscles indicated with lines [12] (reproduced with permission of the illustrator, Sentiel Rommel); and (bottom right) a sagittal section of MG and adjacent structures of a humpback whale (*M. novaengliae*); with coronal section of the mammary gland underneath [18]. **b** Ventrolateral view of an in-situ mammary gland of a gravid adult female porpoise, with overlying tissues removed [17] (available via license: Creative Commons Attribution 4.0 International) **c** Inflated MGs of a lactating female porpoise with the extremities of the nipples ligated [13]

**Fig. 2:**
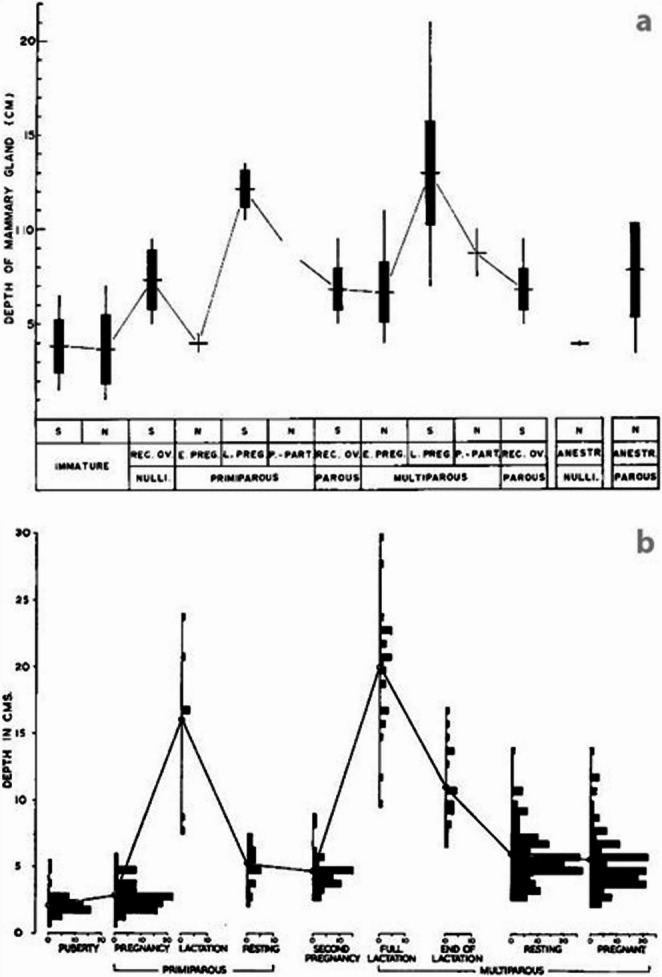
MG thickness (in cm) in cetaceans at various stages of the reproductive cycle, collected during whaling activities. **a**, **b** Frequency distribution of MG thickness of female fin whales (*B. physalus*) [15] and gray whales (*E. robustus*), respectively [16]. Symbols: In **a**, horizontal dashes represent the mean; vertical boxes indicate standard deviation; vertical lines denote range. N: Northbound migrants; S: Southbound migrants

The mammary glands in most marine mammals are found in the mid-caudal abdomen, except in sirenians (dugongs, manatees), whose mammary glands are positioned in the axillae [12]. Cetaceans have long, flattened, and slender paired mammary glands, located longitudinally along the body in both sides of the genital slit (Fig. 1; mammary glands in all figures abbreviated MGs) [12, 13]. The mammary gland bulk tissue (parenchyma) is divided by connective tissue septa into lobules, feeding into common principal ducts, which extend off each gland and connect to the nipples (Fig. 1b). The nipples are located at the caudal ends of the glands, recessed below the mammary slits positioned on each side of the genital slit (Fig. 1c) [6, 8]. The presence of mammary slits can therefore help to determine the sex of a cetacean, although mammary slits have also been described in males of some species [12]. The duration of lactation varies considerably among cetacean groups: mysticete whales typically lactate for 5–7 (maximum 12) months, while odontocetes have longer lactation periods, commonly lasting 1–4 years [8, 14]. In late-pregnant and lactating females, as mammary glands increase in size, they are visible externally as swollen areas cranial to the genital groove [7–9] (Fig. 1c). The thickness of mammary glands has also been shown to vary with reproductive history in humpback (*Megaptera novaeangliae*) and gray (*Eschrichtius robustus*) whales, with lactating multiparous females (those who have borne more than one calf) having thicker mammary glands than primiparous females (those giving birth for the first time) [15, 16] (Fig. 2).

Despite the knowledge of general anatomy of cetacean mammary glands, very few detailed depictions exist. In 1840, Cooper [13] described the mammary glands of a porpoise (*Phocoenidae*) carcass, which were dissected, inflated, and tied at both nipples to characterize the three-dimensional anatomy (Fig. 1c). With this method, Cooper depicted, probably for the first time, the locations of the mammary slits flanking the vaginal and anal slits (Fig. 1c). He also illustrated the slits, both slightly and completely open, exposing the nipple and its orifice [13]. Surprisingly, this work from nearly 200 years ago remains the most detailed visual depiction of cetacean mammary gland morphology and position. Regarding pregnant females, the most detailed image of an active mammary gland remains that of Hepburn from 1893 [17] (Fig. 1b), which illustrates the gland’s anatomic location in a gravid porpoise (*Phocoena phocoenacommunis*), with the principal duct enlarged at its posterior end to form a milk reservoir (lactiferous sinus) (also mentioned by [13]), projecting off the distal end of the gland and communicating to the exterior by the nipple. In contrast, many later studies only provided textual descriptions of gland anatomy, while illustrations, if present, only vaguely indicated topographical location [7, 12, 13] or offered just a generalized picture of internal structure [7, 13, 18] (Fig. 1a).

Although anatomical descriptions of the mammary glands of adult and lactating animals exist in the literature, little is known about the developmental biology of these tissues in cetaceans. Most research on cetacean pregnancy has focused on reproductive physiology and fetal development, while only a few studies have depicted or described in detail how mammary gland morphology is altered during different reproductive stages [6, 7, 13, 17]. Indeed, there are no exhaustive data on embryonic mammary morphogenesis or on macro- and microscopic characteristics as glands mature. However, a general picture of cetacean mammary gland structure and variation can be cobbled together from multiple sources and species.

Histologically, the mammary glands of cetaceans do not differ from those of other mammals; their larger size is not due to a unique tissue architecture, rather an increased number of alveoli per lobule and number of subdivisions per gland lobe [9]. In whales, as in other mammals, immature mammary glands are poorly developed, dominated by (non-glandular) connective tissue with limited vasculature and ducts, and small-diameter glandular lobules [9, 19]. In sexually mature individuals, mammary glands are assigned to four different stages: virgin (young adults with no pregnancies, where lobules are better developed than in the immature stage but less so than in resting stage mammary glands); intermediate (either just before lactation, when lobules start to enlarge, or after, when lobules start to regress); lactating (swollen lobules filled with droplets of actively secreted milk); resting (between pregnancies, when the mammary gland has involuted completely, with a larger quantity of smaller lobules) [9]. Mammary gland involution after weaning remains particularly unexplored, lacking histological or molecular analysis to describe the mechanisms of large-scale tissue remodelling or regression that must accompany the observed changes in gland size. Only one study, describing the mammary glands of blue and fin whales, mentions that although mammary glands develop rapidly at the end of pregnancy, involution after lactation is very slow [9]. Cape fur seals have been shown to delay involution during long foraging trips through a comparatively low concentration of α-lactalbumin, a milk protein typically responsible for mammary epithelial cell apoptosis and thereby the regulation of involution [20]. It is unknown whether similar adaptations of involution control exist in cetaceans.

Most quantitative descriptions of cetacean mammary glands (e.g. gland morphometrics, mass) date back more than 100 years, and focused mainly on large whales, as these were the species obtained through whaling activities (Table 1) [9, 15, 18]. Despite the impact on whale populations, commercial whaling offered unique insights into whale reproductive physiology. For example, scientific reports obtained from these activities correlated fetus length with thickness of mammary glands in some of the whale species caught (humpback, blue, and fin whales), suggesting that as the fetus grew larger, the thickness of mammary glands decreased [21].

**Table 1:** Compilation of mammary gland dimensions of lactating cetaceans. Literature references for body length at sexual maturity and for mammary gland dimensions differ; both are noted in brackets on the Refs column, body length in the left brackets and mammary gland dimensions in the right

Species	Body length at sexual maturity (cm)	Mammary glands				
		Length (cm)	Width (cm)	Thickness (cm)	Weight (g)	Refs
Blue whale (*B. musculus*)	2100–2300	150–200	65	20–30	56,250	[8, 9, 22]
Fin whale (*B. physalus*)	22,500	150–200	65	20–30		[8, 9, 15, 23]
Humpback whale (*M. novaeangliae*)	1202	170	45		17,700	[8, 18, 24]
Gray whale (*E. robustus*)	1170				115,000	[8, 16, 25]
Sei whale (*B. borealis*)	1410			13		[8, 26]
Common minke whale (*B. acutorostrata*)	890			10		[27]
Southern right whale (*E. australis*)	1250			25		[8, 28]
Dwarf sperm whale (*K. sima*)	215	50–52	4	3		[29, 30]
Long-finned pilot whale (*G. melas*)	>375	45	15	7.5	6000	[29, 31]
Common dolphin (*D. delphis*)	155–190	31	7	2	570	[29, 30]
Harbor porpoise (*P. phocoena*)	147	30.5	7.5	2.3	620–700	[8, 32]
Spotted dolphin (*S. frontalis*)	183–213	27		2.8		[33, 34]
Spinner dolphin (*S. longirostris*)	165–170	27		2.8		[34, 35]
Pantropical spotted dolphin (*S. attenuata*)	163–167	27		2.8		[8, 36]

**Fig. 3:**
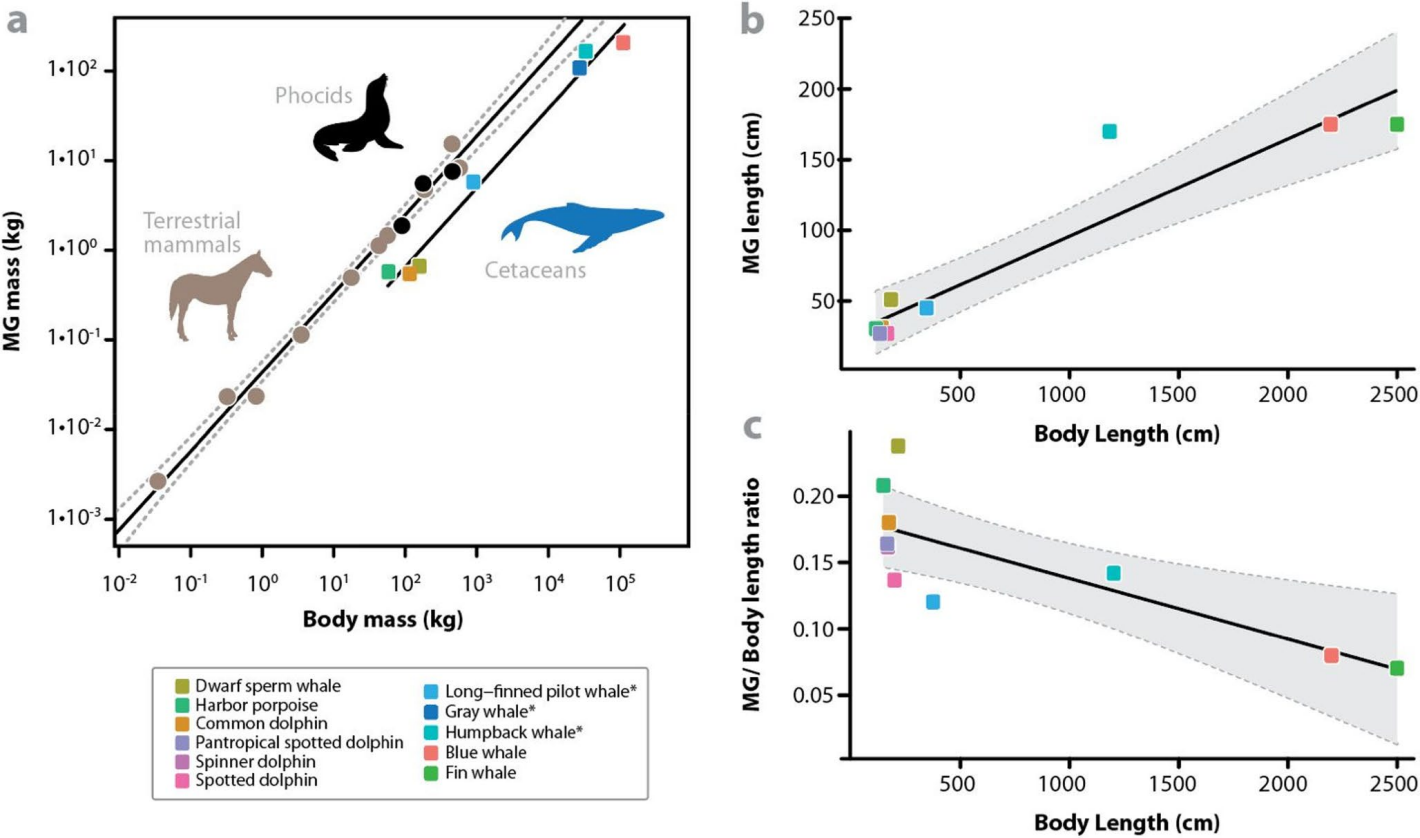
Relationship between body and mammary gland mass and length of different mammals. Each cetacean species is represented by a colored square; the legend for each species is located at the bottom left of the graph **a** Mammary gland mass (MGM) plotted against body mass (BM) on a log10 scale, modified from [8], used with permission from Springer Nature (Licence number: 6135510962304). Black circles represent phocids (hooded seal, harbor seal, and Weddell seal), brown circles, terrestrial mammals, and squares, cetaceans. MGM for cetacean species with asterisks calculated using linear gland dimensions. Regression equations: Terrestrial mammals: Log MGM (kg) = 0.886 * Log BM (kg) − 1.338 (r² = 0.990). Cetaceans: Log MGM = 0.902 * Log BM − 1.965 (r² = 0.983) **b** Linear regression between mammary gland length (Y axis) and body length (X axis) of different cetacean species, based on data from Table 1. Plot generated with R studio version 4.4.1 **c** Linear regression between mammary gland length/body length (Y axis) and body length (X axis) of the same cetacean species as in Figs. 3a and b, based on data from Table 1. Plot generated with R studio version 4.4.1

In a more recent study, a correlation between mammary gland mass and linear gland proportions was suggested, based on data from fisheries-caught/stranded smaller whales and dolphins such as the common dolphin (*Delphinus delphis*) and harbor porpoise (*P. phocoena*), and blue (*Balaenoptera musculus*) and dwarf sperm (*Kogia sima*) whales [8] (Fig. 3a). From our synopsis of the limited available data (Table 1), whereas mammary glands are longer and more massive in larger cetaceans (Fig. 3b), when plotted as a ratio of body length, they are relatively shorter in larger animals (although data from medium- and large-bodied cetaceans are comparatively lacking) (Fig. 3c). However, more detailed descriptions of and correlations among gland and body morphometrics for different species and at different life stages are needed to understand gland physiology (e.g. using the modern diagnostic imaging methods described below).

## Common Methods To Assess the Mammary Gland Morphology and Physiology

In the following sections, we describe current standard methods for physical assessment, laboratory examinations, and diagnostic imaging techniques to evaluate mammary glands. In humans, regular check of these organs is vital for prevention, early detection and treatment of pathologies such as breast cancer [37]. For veterinarians, maintaining udder health in dairy animals is vital for optimal sustained milk production (galactopoiesis), as mammary gland inflammation (mastitis) can affect not only the overall health of the animal, but also milk yield, resulting in economic losses [38]. In contrast, clinical examination methods are limited in marine mammal medicine due to the animals’ aquatic environment and transportation difficulties, with a consequent lack of standardized approaches in these species. Moreover, mammary tumorigenesis in marine mammals is extremely rare, with only a few cases reported in beluga whales (*Delphinapterus leucas*) [39], suggesting a low incidence or underestimation of such neoplasms compared to humans and domestic mammals [40]. As a result, for species under human care (e.g. in zoos and aquaria), mammary glands are typically ignored in clinical examinations, only receiving focused evaluation when they are visibly swollen in association with pregnancy or pathology (pers obs., Sánchez-Contreras).

### Laboratory Investigations

Mammary glands are under hormonal control for their development and growth, especially during important ontogenetic stages like lactation. Although multiple hormones regulate aspects of milk production (e.g. oxytocin, progesterone, estrogens), prolactin is the most important [41]. Prolactin can promote mammary gland growth, initiate the production of milk (lactogenesis), and sustain lactation (galactopoiesis) with the help of other metabolic, local, and reproductive processes [42]. Baseline concentrations of prolactin fluctuate depending on the reproductive stage of species such as humans, rodents, and ruminants [43]. In a diversity of mammals (from humans [44] to bottlenose dolphins [6]), prolactin concentrations rise with the progression of pregnancy and reach their highest concentrations in the last month before parturition. In marine mammals, prolactin is known to be of the utmost importance for the development of mammary gland secretory cells, for example, increasing in otariids (eared seals) usually one or two days before parturition and showing the highest levels ~ 0–3 days postpartum [45]. Measuring this hormone is therefore extremely valuable for monitoring the development of important reproductive events such as pregnancy and lactation, which can help differentiate healthy and unhealthy pregnancies and predict successful lactation, which is vital for offspring survival.

Blood is the most common biological sample taken for measuring prolactin levels in humans and other animals [46, 47]. Prolactin levels are affected by age, sex, stress, circadian rhythm, and even drug ingestion, and therefore need to be considered when measuring hormone concentrations [48]. Medical and veterinary researchers have sought less invasive methods for measuring prolactin, in urine [49, 50] and saliva from humans, dogs, and rhesus macaques (*Macaca mulatta*) [51–53]. To our knowledge, only urinary analysis has been used in marine mammals as a non-invasive method to determine prolactin levels, but only in bottlenose dolphins [6] and belugas [54] under human care. In fact, whereas other reproductive hormones (e.g. progesterone, estrogen) are regularly monitored in managed animals during pregnancy [54–56], prolactin is not commonly measured in marine mammals, in part due to the lack of specific essays for these species, limiting understanding of normal ranges of this hormone and its role in reproduction. Veterinarians and researchers would therefore benefit greatly from better knowledge of baseline ranges of prolactin in healthy female cetaceans of various ages and reproductive stages, to predict optimal mammary gland development for lactation and calf survival.

Other factors surely regulate mammary gland development (e.g. the milk protein α-lactalbumin, mentioned above; [20]) and therefore could prove useful for regular diagnostics, but are yet to be thoroughly explored for cetaceans. Placental lactogens, for example, are peptide hormones produced and secreted by the placenta [57, 58], identified in both primates and non-primates, including rodents [58, 59] and ruminants (cows, goats, and sheep; [60, 61]. These hormones serve key regulatory roles throughout pregnancy, promoting fetal and placental growth [60, 62] and facilitating development and functionality of the mammary gland during and after gestation. Even though growth of mammary ducts is primarily regulated by ovarian steroids, complete lobuloalveolar growth is only fully achieved with lactogenic hormones, like placental lactogens [61]. Placental lactogen concentrations fluctuate throughout pregnancy [61], with concentrations positively correlating with placental mass in humans [59], the stage of gestation in cows and sheep, and fetal number/milk production in sheep [60]. In humans, after the placenta is delivered, this hormone is quickly cleared from the maternal bloodstream [62]. In cetaceans, placental lactogens were identified immunohistochemically in three rorqual whales: Bryde’s (*Balaenoptera brydei*), sei (*B. borealis*), and common minke (*B. acutorostrata*) [63]. Localization of the hormone to placental trophoblast cells in these species suggest a primary secretion in the maternal bloodstream, yet the precise effects of placental lactogens on either mother or fetus remain uncertain.

### Physical Assessment

The most common way to evaluate mammary gland health in humans and other mammals is physical examination. In humans, as part of an integrative assessment, a clinical breast exam (CBE) provides vital information for diagnosis of breast diseases and abnormalities [64] and can aid in early detection of breast cancer as a complement to mammography [37]. The basic components of a CBE include visual inspection and palpation of the breasts and nipples, characterizing various parameters such as color, symmetry, shape, temperature, texture, venous patterns, and size and allowing detection of lesions or masses [37, 65, 66]. Although CBE can successfully detect breast cancer without additional tests, multiple factors relating to the patient and examiner can impact examination sensitivity and specificity [37]; as a result, CBE has been increasingly conducted alongside modern diagnostic imaging tools (see below), particularly for diagnosing breast malignancies [67, 68].

In veterinary medicine, physical assessment of the mammary gland has been performed mainly in farm animals such as cattle and to a lesser extent for small ruminants [69–71]. As in human CBE, physical examination of the udder and teats in veterinary medicine includes visual inspection followed by palpation, allowing inexpensive and quick early detection of pathologies such as mastitis or intramammary infections [69]. Examinations describe a diverse range of features that speak to the health of mammary glands: the texture, volume, symmetry, and thickness of the teat and nipple orifice; udder shape and edema; mastitis, knotty tissue; as well as presence of wounds, inflammation or swelling of the teat (thelitis) and inflammation of the teat cistern (cisternitis). Microbiological tests and milk somatic cell counts (MSCCs) are often used in follow-up to verify the presence of infections [69, 70]. These evaluations are not only useful for determining herd health with regard to milk production [72], but can also aid in identifying inadequate use of milking equipment that could damage mammary tissues (e.g. in dairy goat farms) [73].

In marine mammals, only a few descriptions of physical evaluation of mammary glands exist and only for species under human care, such as polar bears (under anesthesia) [74]. The limited adoption of this approach is due in part to challenges faced by the habitats and anatomy of species. In captive dolphins, for example, the blubber and the internal positioning of the glands makes palpation largely ineffective as a diagnostic technique. As a result, physical evaluation of mammary glands is performed only when the animal’s external visual appearance indicates the presence of infection. In these cases, when animals are lactating, milk samples might also be taken to verify infection [75, 76]. For these reasons, imaging techniques such as ultrasound (see below), are particularly important complements for laboratory analyses in marine mammals.

### Imaging Techniques

Across species and medical contexts (human or veterinary), the most common imaging techniques used for mammary gland assessment include radiography (mammography), magnetic resonance imaging (MRI), positron emission tomography (PET), computed tomography (CT) scanning, and ultrasonography [11]. Because of its comparative portability and ability to render soft tissues in real-time, ultrasonography is particularly apt for mammary gland imaging and so we devote more time to discussing its different modalities and future applications.

Each method described below is apt for imaging particular tissues, creating visual contrast based on characteristics of tissue composition and structure, whether healthy or pathological [11, 77]. As a result, the properties of tissues also affect their imaging: in humans, age, hormones and the use of prescription drugs can cause tissue changes (e.g. increased fibrosis) that limit imaging and hamper the diagnosis of breast pathologies. Imaging techniques, therefore, are usually considered complementary to visual and physical assessments as diagnostic tools for detecting abnormalities or monitoring pathology progression in mammary glands [78]. Depending on the findings, laboratory tests might still be necessary companions to imaging to reach a definitive diagnosis [79–82].

#### Mammography

Mammography is one of the most common imaging techniques used in human medicine to assess women’s breast tissues. This radiographic method creates an X-ray shadowgram by irradiating the breast, compressed onto an underlying platform, above an X-ray sensitive film or receptor, where the X-ray tube can rotate to create specific radiographic projections from diverse angles [77, 83]. To detect abnormalities (e.g. micro-calcifications, masses), high resolution and low contrast are needed with a low dose of radiation to minimize patient exposure risk [84]. Compression of the breast during the examination is important to lower the dose of radiation, enhance contrast, avoid movement artifacts, and minimize breast tissue overlap (superimposition), which can hamper assessment and pathology localization [77]. Based on image grayscale variation (a function of radiodensity), mammography can detect pathological soft tissue alterations, calcifications of different shapes and sizes, as well as disruptions in breast architecture [77]. Moreover, this imaging technique is efficient for early cancer detection, reducing mortality and helping improve treatment outcomes [85, 86]. Risks associated with this technique, such as radiation exposure, have dropped significantly due to the development of full-field digital mammography [84], where an electronic detector is used to absorb the radiation applied to breast tissues [83, 87]. In contrast to traditional film mammography, digital processing methods can be applied after image acquisition to modify brightness and contrast without the need to expose the patient multiple times [87]. In veterinary research and practice, mammography is applied mainly in preclinical settings (e.g. animal subjects used for human medical research) and not as a diagnostic tool in a veterinary clinical setting. For example, studies have examined the mammary glands of mice in-vivo [86] or udders from slaughtered sheep [88] to improve mammography in human patients.

**Fig. 4:**
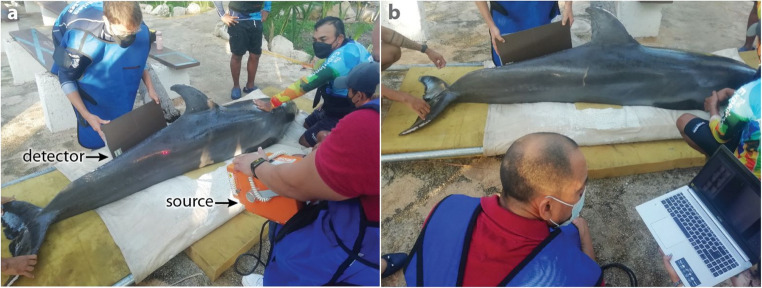
Field-based plane X-ray procedure for a male Atlantic bottlenose dolphin to evaluate the spine. **a** The dolphin is positioned for imaging, with two trainers stabilizing it while two veterinarians conduct the procedure. The veterinarian in the red shirt holds the X-ray source while another veterinarian holds a digital detector on the contralateral side of the animal. **b** The veterinarian analyzes the digital X-ray image displayed on a laptop. Photo credit: The Dolphin Company

In marine mammal medicine, mammography is not feasible, due to the internal and ventral abdominal location of the glands (Fig. 1), which prevents isolation of the mammary glands within the scanning field. However, with the development of portable and wireless X-ray machines, radiographic studies have been successfully performed in live marine mammals for skeletal, soft tissue and intraoral examinations (e.g. Figure 4) [89, 90]. The same risks and limitations associated with radiological exams exist for marine mammals, but in some cases these species present even greater challenges: given their reproductive anatomies and aquatic habitats, patient movement, correct positioning of structures of interest, and equipment damage (e.g. from salt water) are all relevant obstacles [89].

#### Computed Tomography (CT) Scanning

Like mammography/plane radiography, computed tomography measures the differential attenuation of X-ray beams as they pass through various tissues. A CT scanner involves a circular rotating structure (gantry), which holds the X-ray source and associated radiation detectors on opposite sides of the patient, as well as a moving table that passes through the gantry where the patient is lying [89]. In this way, a CT scanner creates many (often thousands of) radiographic projections taken from multiple positions around the subject to reconstruct tissue arrangements in 3D, enabling cross-sectional and volume renderings of anatomical features and differentiation among diverse tissue types based on their radiodensity [91]. CT is widely utilized in human medicine to evaluate both normal and pathologically altered organs, particularly to image tissues with high attenuation (e.g. bones, teeth) or soft tissues injected with contrast media (e.g. mammary gland vasculature) [92]. Compared to MRI (see below), the achievable resolution is similar, but CT is considerably faster [91, 93]. The application of CT in breast tissue evaluation in women has been limited, primarily due to concerns of high radiation exposure (e.g. to organs in addition to the mammary glands); as a result, mammography has remained the preferred screening method [92, 94, 95]. However, several studies have focused on developing CT technology specifically for breast imaging (e.g [96–98]). , which can be particularly effective for elements with high attenuation, such as microcalcifications and neoplastic soft tissue elements, where CT imaging can deliver excellent anatomical detail and volumetric imaging [94].

The increasing accessibility of CT scanning, its lower cost relative to other advanced imaging techniques, and the availability of free viewers for DICOM (Digital Imaging and Communications in Medicine) files, have facilitated the adoption of this modality in veterinary medicine [91, 99]. CT has been used to examine a diverse range of species, encompassing companion animals, wildlife, and even aquatic species, and to evaluate various anatomical elements, including the thoracic and abdominal cavities, central nervous and musculoskeletal systems [91, 100]. The broadened interest in this imaging modality and in digital imaging approaches has also supported a rise in online imaging databases (e.g. *Morphosource.org*, including the *openVertebrate* project: https://www.floridamuseum.ufl.edu/overt/*)*, often geared toward skeletal research, where comparative anatomical data can be flexibly shared and explored. However, the same attention has not been given to mammary glands, with existing research primarily focusing on dairy species such as goats and cattle. In goats, CT has allowed the visualization of udder substructures and surrounding fascia and muscles. Moreover, visualization of the mammary vessels after the injection of contrast medium was also possible, allowing different areas of the lactiferous sinus to be identified and their volumes calculated non-invasively [92]. Additionally, CT imaging helped distinguish between tissue types in dairy heifers undergoing somatotropin treatment [101], successfully differentiating between parenchymal and extraparenchymal tissues [101, 102]. This enabled non-destructive cross-sectional and volumetric measurements of the mammary gland, which are far more precise and less labor-intensive than previous estimates derived from manual dissections [103].

**Fig. 5:**
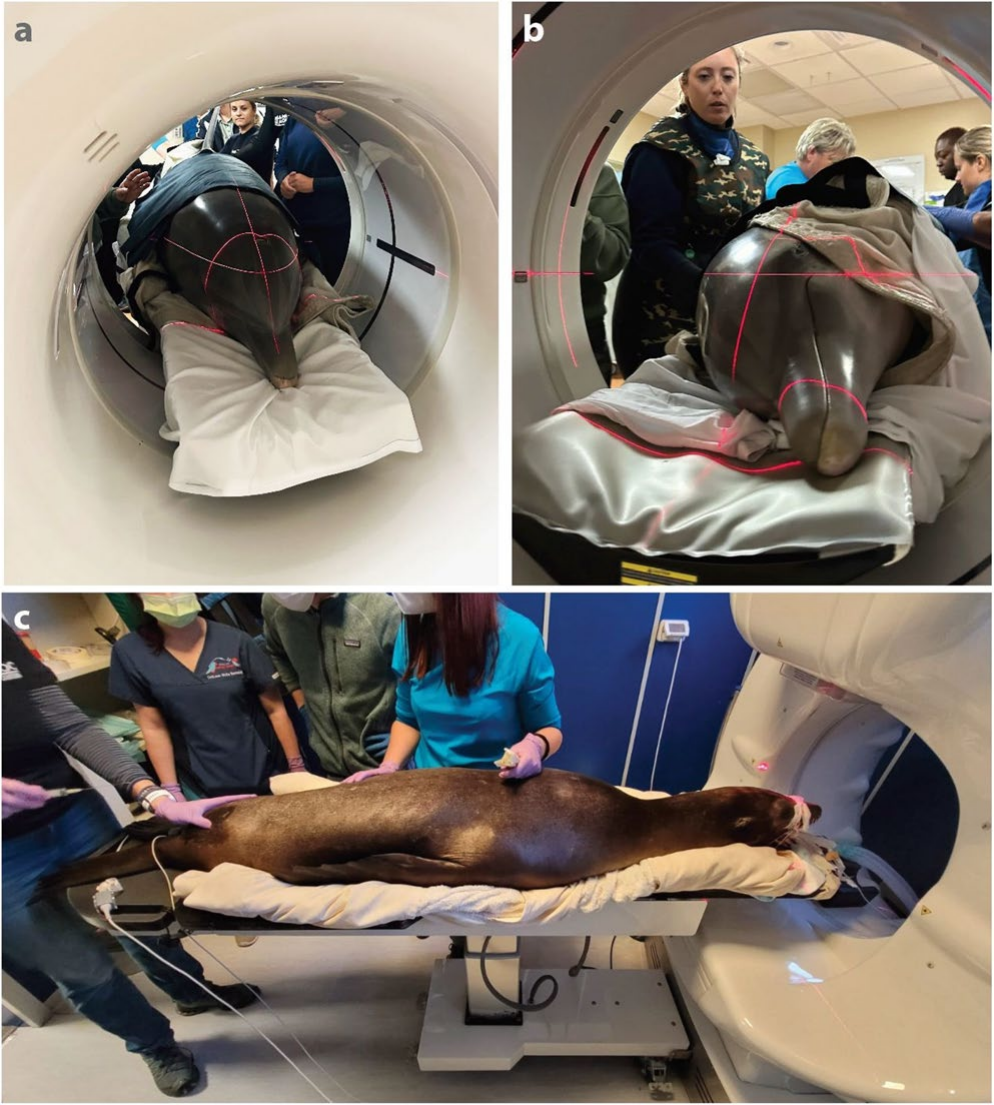
Marine mammals under human care undergoing CT and MRI scan procedures. **a** Female Atlantic bottlenose dolphin, follow-up CT scan to evaluate a respiratory condition (USA). **b** Same female Atlantic bottlenose dolphin as in Fig a but positioned in left-lateral recumbency on the CT table **c** Female California sea lion undergoing an exploratory CT scan for a pancreatic condition (Italy). Note the limited diameter of the CT scan bore/gantry, which restricts scanning of larger animals and/or species with unwieldy appendages (e.g. dorsal fins). Spatial limitations and scanning table procedures are similar for MRI scans. Photo credit: The Dolphin Company

**Fig. 6:**
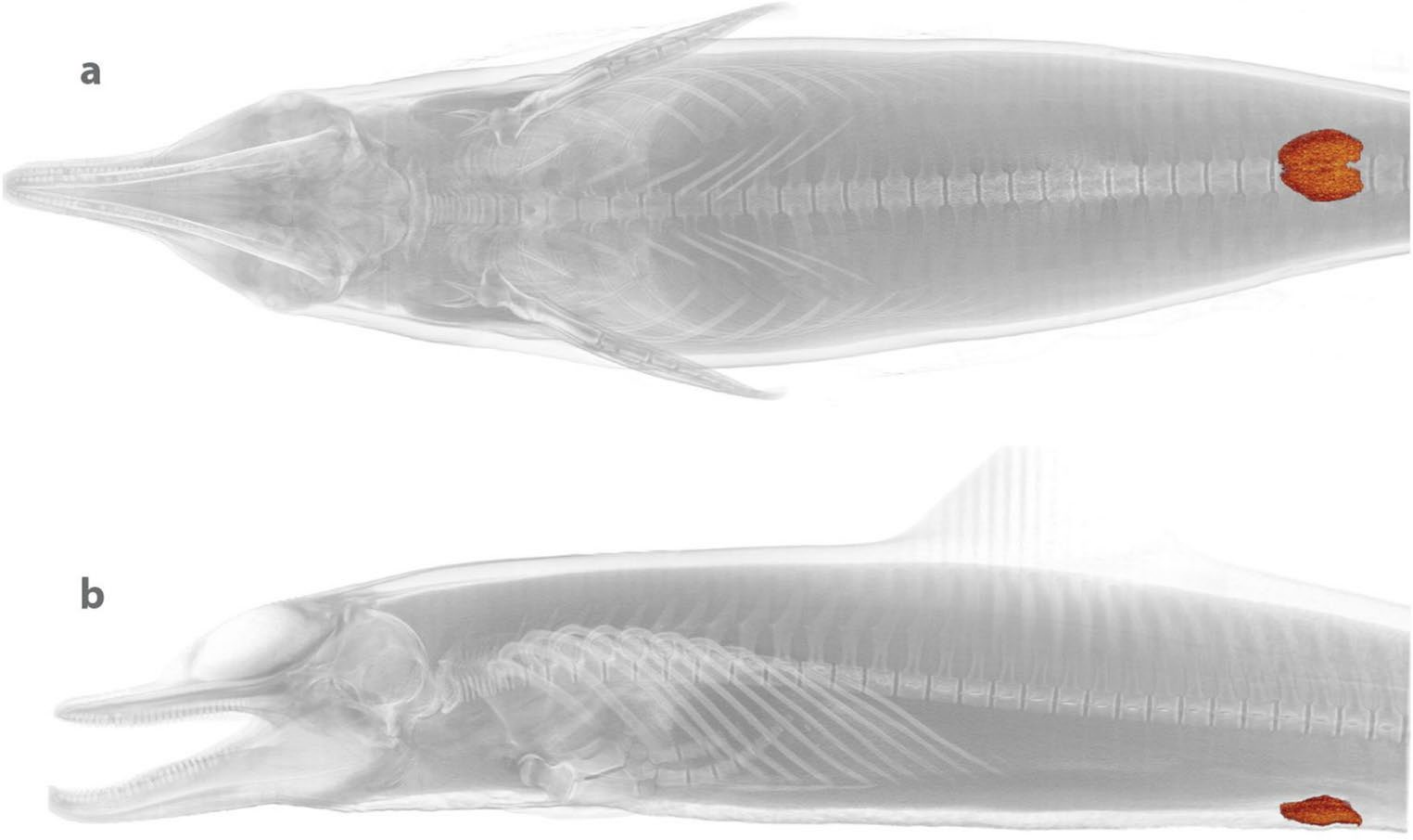
3D reconstruction of the MGs (highlighted in red) from a PMCT scan of a Code 2 Indo-Pacific bottlenose dolphin carcass; scan data have been thresholded to show the skeleton and the MGs segmented and volume-rendered by the authors using Amira software. **a** Ventral view, showing left and right MGs, located above the genital region and below the lumbar vertebrae. **b** Left lateral view, with the caudal end of the MG oriented towards the mammary slit, indicating the location of the nipple; compare with compiled anatomical illustrations in Fig. 1. In contrast to available imaging data for other anatomical regions, as far as we are aware, this is the first 3D digital rendering of a cetacean MG, underlining the need for more data on these organs. PMCT scan provided by Ocean Park, Hong Kong

In marine mammal medicine, CT has already been employed in various experimental and anatomical studies of live animals: for example, to investigate body composition (blubber, skeletal muscle) in grey seal pups (*Halichoerus grypus*) [104] and measure splenic volume in harbor seals (*Phoca vitulina*) and California sea lions (*Zalophus californianus*), either restrained, sedated, or anesthetized for the procedure [105] (Fig. 5). Although plane radiography remains a more standard approach, CT scanning has shown great utility in marine mammal medicine by offering superior imaging detail and the ability to visualize complex structures in 3D (i.e. without the superimposition of 2D projections), improving the evaluation of disease severity and surgical approach planning [106]. In a clinical context, CT scans of marine mammal patients have revealed various conditions that are undetectable through common diagnostic methods or palpation [107], for example, diagnosing diseases located deep within the animal’s body (e.g., such as bone lesions) [106]. In addition, advancements in CT technology have been especially beneficial for studying marine mammal anatomy [108], particularly head and brain structures [109, 110], as well as sensory organs linked to feeding strategies [111]. Particularly for marine mammal medicine, CT scanning has proved helpful in postmortem detection of pathologies difficult to identify in conventional radiographs or ultrasonography [89]. The application of postmortem CT scanning (PMCT) in cetaceans has therefore grown over the years, used by marine mammal veterinarians and stranding response programs [112]. The non-invasive nature of CT is a great advantage in this context: for example, researchers have achieved precise 3D reconstructions of internal organs in an Indo-Pacific bottlenose dolphin just hours after the animal’s death (Carcass Condition Code 2) [113] (Fig. 6). Furthermore, PMCT has demonstrated significant utility in identifying mammary gland ducts and parasitic cysts in deceased stranded Indo-Pacific finless porpoises (*Neophocaena phocaenoides*) despite the advanced state of decomposition of the carcasses [114]. In these examples, CT not only allows accurate depiction of the exact 3D locations and orientations of various anatomical structures, but also quantification of morphology (e.g. gland volume).

It is worth noting that, beyond higher costs relative to other imaging techniques, the logistical issues of CT scanning marine mammals remain tremendous, from transporting animals to the CT scanner, to finding machines that can accommodate the impressive bulk of marine species; for example, the adult female Atlantic bottlenose dolphin in Fig. 5a weighed 175 kg, despite being small enough to fit through a medical CT scanner designed for humans. Furthermore, marine mammal gross morphology is often not compatible with scanners designed for human patients: the length of the body and especially the dorsal fin (in some cetaceans) can pose challenges for animals to fit CT machines’ internal gantries [89]. Moreover, the health risks involved in handling the animals out of the water —respiratory compromise, handling stress, loss of thermoregulation [107]— greatly restrict possibilities for regular or repeated in vivo CT imaging (e.g. for longitudinal studies of organ growth). These challenges help to explain why CT has yet to be utilized explicitly to evaluate the mammary glands of live marine mammals.

#### Magnetic Resonance Imaging (MRI)

Like CT, MRI permits three-dimensional reconstructions of anatomy, allowing virtual (and therefore non-destructive) dissection in any sectioning plane [115]. MRI, however, does not rely on ionizing radiation, but rather generates images based on how protons in different tissues respond to induced magnetic fields [116]. As a result, MRI provides better visualization of soft tissues than X-ray based imaging methods [117], and is therefore the preferred method to study the central nervous system for clinical work [89] (although contrast agents like iodine are increasingly used to enhance tissue contrast for CT study of deceased specimens/tissues; [118, 119]). For mammary gland analyses, MRI provides a higher spatial resolution (1 mm) than ultrasonography (albeit typically not better than CT) [11]. This and MRI’s superior soft tissue contrast allow it to detect mammary gland cancers not recognizable by other imaging modalities (e.g. invasive ductal carcinoma) [120]. Additionally, hardware attachments for organ-specific examinations (e.g. breast coils) can improve diagnostic quality, by spreading tissue to better observe abnormalities and prevent motion artifacts produced by respiration [121, 122]. As a result, MRI is nearly 100% accurate in identifying breast masses in women compared to other diagnostic modalities, such as mammography and physical assessment [123].

MRI’s high sensitivity in lesion detection is due to its ability to apply different methods of signal contrast generation for image acquisition [121]. T1-weighted (T1-w) and T2-weighted (T2-w) imaging techniques exploit different aspects of how water molecules in tissues respond to magnetic field pulses, converting these variations into grayscale intensity differences [124]. As a result, T1-w and T2-w images can effectively distinguish different tissue types: whereas T1-w images render healthy soft tissues and fat bright and fluids appear dark, T2-w images enhance fluid signals and highlight abnormalities such as inflammation [124]. Diffusion-weighted imaging (DWI), on the other hand, measures random water movement within tissues, reflecting how tissue architecture facilitates or restricts fluid transport; for example, since higher cell densities reduce water diffusion, DWI is therefore particularly useful for detecting cancers [121, 125].

MRI relying on a different imaging modality than CT (i.e. radio waves rather than X-rays) means that the limitations of the technique also differ. Assessing a specific body region typically requires multiple image types taken at each projection angle, resulting in a relatively longer scanning time compared to CT scans. Additionally, the presence of gas within anatomical structures can produce artifacts that may mask or simulate certain pathologies. Systems or regions that naturally contain gas, such as the respiratory and gastrointestinal tracts, are therefore often better evaluated using CT or radiography [89]. Moreover, the strong magnetic field generated by MRI can react strongly with metal objects; as a result, patients with metal implants in their bodies are typically forbidden from entering MRI machines [115]. Besides causing severe image degradation, ferromagnetic structures inside the body can be attracted to the magnetic field generated by the MRI machine, displacing the object, and resulting in tissue damage or even death [99].

MRI scanners are increasingly accessible to veterinarians through both human and veterinary hospitals, as well as mobile units. This has led to a greater utilization of MRI for evaluating neurologic, orthopedic, oncologic, and ophthalmological disorders across animal species [99]. Despite its suitability for imaging soft tissues, MRI has been used to a lesser extent in assessing the mammary glands of companion and farm animals. In companion animals, MRI protocols designed for mammographic studies have been used with specially designed coils to study mammary gland tumors in dogs, successfully characterizing morphological aspects and angiogenesis of malignant tumors using conventional and contrast-enhanced MRI, respectively [126]. Regarding farm animals, udders from live lactating goats [127] and slaughtered nulliparous, non-pregnant dairy heifers [128] have been analyzed using this modality to determine baseline mammary gland composition and volume. Mammary gland imaging often requires euthanasia in large farm animals, which typically exceed the capacity of MRI machines [128]. This limitation significantly restricts the use of MRI for studying mammary gland structures and functions in these species. Similarly, the application of MRI in marine mammal medicine, including the evaluation of mammary glands, has been limited due to the size and weight constraints of these animals, as well as the requirement for patient stillness during live animal imaging. Instead, MRI has been most successfully employed in marine mammal research on post-mortem specimens, particularly stranded wild individuals, to study anatomical structures such as the brain [129, 130] and to determine the cause of death prior to necropsy [131]. In this regard, the non-invasive aspect of MRI has proved particularly useful to marine mammal biology, by providing valuable anatomical information without specimen damage, capturing delicate anatomical features before they degrade after death [132].

#### B-mode Ultrasonography

**Fig. 7:**
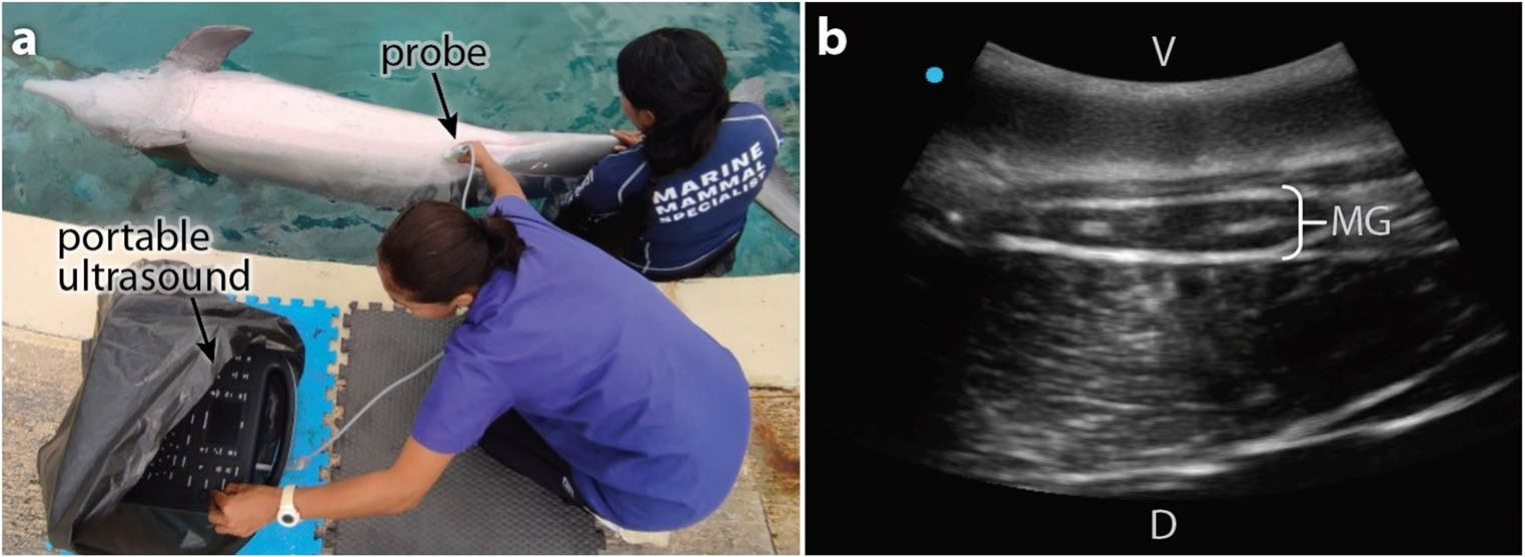
Ultrasonographic evaluation of the mammary glands in a female Atlantic bottlenose dolphin **a** Marine mammal veterinarian performing a pool-side ultrasound exam on the right mammary gland of the dolphin, positioned in dorsal recumbency by its trainer. The portable ultrasound machine is covered by a plastic bag to avoid water damage. **b** B-mode ultrasound scan of the dolphin right mammary gland, in transverse view. MG = mammary gland, V and D = ventral and dorsal aspects of the body, respectively. The blue dot on the top left of the ultrasound scan shows the direction the orientation marker, a position indicator on the probe, is pointing.

Ultrasonography uses a transducer to transmit ultrasound waves into body tissues; some waves (echo signals) return to the transducer to generate real-time, two-dimensional images, while others continue travelling deeper into the body [133, 134]. The strength of these echoes, captured by the transducer, determines the brightness (B-mode) of the displayed image, allowing for a grayscale representation of the tissue. The brightness of the ultrasound images reflects a combination of the characteristics of the ultrasound equipment itself and, most importantly for diagnostics, how different tissues reflect or absorb ultrasound waves [135, 136], with factors such as tissue density influencing the resulting image. For its ease of use and ability to accurately distinguish tissue types, B-mode is the most widely used type of ultrasound imaging (Fig. 7b).

Ultrasound imaging offers several advantages that enhance its utility in various clinical settings. Notably, it eliminates radiation exposure, provides real-time image generation, and is generally more cost-effective than other imaging techniques [133, 137]. Additionally, the portability of ultrasound machines is a significant benefit; relative to CT or MRI, many ultrasound units are comparatively affordable and readily available (e.g. even purchasable from online retailers), easily transportable, and capable of operating on battery power. These devices typically match the size of a laptop [138]. Moreover, wireless hand-held sonographic machines have emerged as practical, fast, affordable, and safe tools [10], particularly in surgical procedures and emergency cases involving trauma, where rapid diagnoses are critical. Furthermore, their use increases access to imaging in remote areas lacking reliable electricity, thereby helping to bridge healthcare and fieldwork gaps in limited-resource settings [10, 139–141].

Pertinent to mammary gland imaging, ultrasound may be less effective in patients with a large proportion of body fat (e.g. whether from obesity or blubber), in cases where anatomical structures of interest are obscured by excessive gas or fluid [133, 138], or due to increased glandular tissue and milk secretion in lactating breasts, which can enhance echogenicity/radiodensity and complicate image interpretation [142]. Ultrasound also has limited penetration depth, especially in denser tissues, which may result in suboptimal image quality, particularly for larger animals and/or deeper anatomical structures [143]; to account for this, time gain compensation (a graded adjustment of signal amplitude) is used to normalize grayscale intensity and gain across 2D images to create signal uniformity in image visualization [144]. On ultrasound machines, this correction can be manually applied to modulate signal strength relative to depth, allowing real-time adjustments based on the properties of the tissues and the organs being imaged [80]. As a result of these limitations, while ultrasound is effective for many clinical applications, it is mainly a complementary rather than a standalone diagnostic method and therefore is often combined with other imaging modalities for a comprehensive evaluation [135]. For example, ultrasound can distinguish between breast cysts and solid masses, and can detect malignancies in patients with dense breast tissue that may be missed on a mammogram [145]. Additionally, recent advancements in ultrasound resolution and tumor classification based on specific sonographic features have significantly improved the ability to differentiate between benign and malignant masses [136, 146].

Ultrasonography is widely used in veterinary practice due to its non-invasive nature and real-time imaging capabilities, and has been employed to investigate morphological and physiological characteristics of the mammary gland in various dairy species, including ewes and heifers [147, 148]. Data provided by ultrasound on the internal architecture of the udder facilitates evaluation of the teat, mammary parenchyma, lactiferous ducts, and gland cistern, as well as identification of vascular anomalies [81, 147–149]. For example, in ewes, ultrasonography has revealed changes in the parenchyma with reproductive status, such as pregnancy and the termination of lactation [149]. B-mode ultrasound has also been used for mastitis diagnosis [150, 151] and to detect small mammary abscesses before they become palpable, as well as to differentiate them from cysts or haematomas [148].

In contrast, ultrasound has barely been used in assessment of the morphology and physiology of cetacean mammary glands, which have instead mostly been characterized by dissection from either freshly deceased animals or biopsies from live individuals. Ultrasound approaches, however, stand to offer more flexible and comprehensive characterization options, especially given the advent of more portable transducers/units (Fig. 7a) [10, 152, 153]. To our knowledge, ultrasound-based research of cetacean mammary glands is scarce: just three studies have used ultrasound to characterize morphological and structural changes in mammary glands across reproductive stages —in Atlantic bottlenose dolphin (*Tursiops truncatus*) [6, 10] and finless porpoise (*Neophocaena asiaeorientalis*) [7]— contributing to a better understanding of reproductive physiology (e.g. demonstrating that mammary glands enlarge and change their morphology at the end of pregnancy [6]). One of these studies [10] also compared the accuracy and efficacy of cart-based and hand-held ultrasound equipment for assessments, providing guidance on scanner choice for studying mammary gland health and morphology in research settings. These studies show the potential of ultrasound, but offer limited windows into the reproductive cycles of two species. Adopting ultrasound as a more standard diagnostic tool and incorporating a wider range of reproductive stages will establish baseline mammary gland morphometrics for cetaceans, while enhancing understanding of the growth and maturation of mammary tissues, for example, in relation to hormonal changes throughout different life stages.

In clinical settings (e.g. animals under human care), cetacean mammary glands are still typically not included in routine ultrasonographic examinations, but rather only examined to confirm diagnosis of specific pathologies, such as parasitic mastitis [75, 154]. The absence of regular assessments of mammary glands in dolphin facilities with breeding programs is surprising, considering the critical role these organs play in calf survival. The superficial position of mammary glands in the caudal abdomen of cetaceans (Figs. 1 and 6) [12], however, illustrates how these organs may be easily incorporated into regular veterinary ultrasonographic evaluations. Naturally, as most ultrasound units are not waterproof, extra care needs to be taken when performing imaging procedures in proximity to the water [10]. Additionally, successful ultrasound examinations require that animals become accustomed to both the probe and the examination process itself (Fig. 7a). Not all marine mammal species, however, can be easily trained for these procedures (e.g. sea lions [155]), complicating routine assessments.

In addition to B-mode ultrasonography, other ultrasound modalities can evaluate different tissue properties, including vascularity, volume, and stiffness. The following sections provide concise overviews within the contexts of human and veterinary medicine.

#### Color Doppler Sonography

The real-time assessment capability of ultrasound is particularly useful for evaluating moving anatomical structures, including muscles, joints, tendons, and ligaments [156–158]. Moreover, ultrasound can be used to assess an organ’s blood flow through color Doppler sonography, where images are generated by waves reflected from the movement of red blood cells inside blood vessels [159]. In color Doppler ultrasound, flow in vessels is represented according to whether the blood is moving towards (blue) or away (red) from the probe and according to the settings established at the start of the examination [160]. In human medical evaluations of mammary glands, this modality has been used to great avail, for evaluating vessel arrangement, blood flow, and the extent of vascularization in women’s breasts [148, 161, 162]; for assessing vascularization of mammary lesions to distinguish between benign and malignant lesions [163]; and to detect inflammatory hyperemia in abscesses and mastitis [164]. In veterinary medicine, color Doppler sonography has been used in dairy animals to document changes during lactogenesis in healthy and diseased udders of ewes [81]; to evaluate blood flow disorders in goats and sheep [148, 149]; and to measure mammary blood flow variability in lactating cows [165]. However, to our knowledge, there is no literature on the use of color Doppler sonography to assess mammary blood flow in cetaceans. Insights gained from such assessment could help determine how mammary blood flow changes during lactation, but also across different reproductive states and mammary gland pathologies, such as mastitis.

#### Three-dimensional (3D) Ultrasound

Because traditional ultrasound only generates 2D images, with the visualization plane a function of probe angle, it requires a skilled operator who can mentally reconstruct an organ in three dimensions in order to interpret output images. In contrast, 3D ultrasound provides real-time 3D representations of anatomical structures, allowing more immediate understanding of organ borders and spatial relationships [166–168]. As a result, 3D ultrasound has been incorporated into diverse medical procedures, especially where precise identification of an anatomical location is paramount, such as in biopsies, drug delivery and detection of contrast agents [169, 170], as well as in reproductive medicine, mainly to locate abnormalities in the ovaries, uterus, and endometrium [168].

3D ultrasound has emerged as a valuable tool for breast examination, allowing detailed examination of tissue architecture, improving characterization of normal tissue and solid masses, thus increasing accuracy in diagnosis [171, 172]. Additionally, automated 3D breast ultrasound systems have streamlined imaging, reducing operator dependency and variability [173], enhancing workflow efficiency in clinical settings, and paving the way for AI-mediated approaches in image evaluation [174]. Furthermore, machine learning-supported automation of image quality assessments can ensure that artifact-free images are available for diagnostic evaluation, improving diagnostic accuracy and minimizing the risk of misleading results [175]. Nonetheless, the cost of implementing 3D ultrasound systems poses a potential barrier to widespread adoption in various clinical contexts [173].

In veterinary medicine, there is limited use of 3D ultrasound to evaluate the reproductive tract, diagnose pregnancy, and perform examinations in zoo animals, companion animals, and equine medicine [176]. In dairy animals, 3D ultrasound has been used to produce high-quality 3D images of the mammary glands and their structure, including the parenchyma and lactiferous ducts [176, 177]. However, to our knowledge, 3D ultrasound has not been used to study or measure mammary gland volume in marine mammals.

#### Shear-wave Elastography (SWE)

Shear-wave elastography is an ultrasonographic technique that induces lateral shear wave movement through acoustic pulses generated by a transducer, allowing quantitative analysis of tissue stiffness for differentiation of healthy and unhealthy tissues [178–180]. Variation in organ stiffness can indicate pathological alterations to soft tissues (e.g. fibrosis associated with cancer, atherosclerosis, and chronic hepatitis) [181, 182]. In point shear wave elastography (pSWE), a transducer emits a focused acoustic pulse that creates small displacements in the tissue within a small region of interest (ROI; typically < 2 mm; [183]), which are then detected by multiple tracking beams. This allows the system to calculate the average shear wave velocity in the ROI, measured in meters per second (m/s) [180, 184]. Importantly, the operator must select a uniform and healthy tissue as a reference material for comparison with the ROI to distinguish between benign and malignant lesions. In contrast, two-dimensional shear wave elastography (2D SWE) induces shear waves across multiple focal zones simultaneously, measuring the speed of shear waves as they propagate throughout a larger area. Data is then integrated across the entire ROI and displayed as a stiffness color map, with areas of high and low stiffness shown in red and blue, respectively. In this way, elastography provides real-time visualization of tissue elasticity for immediate assessment and decision-making for diagnosis and treatment, thereby also minimizing the need for surgical biopsies [184–186]. Nonetheless, the technique is limited in evaluation of tissues that are deeper or shielded by fat layers, where acoustic shadows can affect image quality [184]. Moreover, patient positioning, movement (e.g. breathing), and tissue characteristics can introduce variability in stiffness measurements, complicating standardization [181]. Accordingly, as with other ultrasound techniques, elastography should be used in conjunction with other imaging modalities and clinical findings, as it may not provide definitive diagnoses on its own [183].

In human medicine, shear-wave elastography has been primarily applied to evaluate liver fibrosis and, more recently, to study tissue stiffness in the prostate, kidney, thyroid, lymph nodes, muscles, and breast (reviewed in [184]). Similarly, in veterinary medicine, this technique has been used to assess normal soft tissues in dogs, differentiate between benign and malignant conditions of lymph nodes and mammary glands, and evaluate liver stiffness changes following radiofrequency ablation [180, 187, 188]. In cats, it has helped characterize the normal appearance of the kidneys, liver, and spleen [189]. However, to our knowledge, shear-wave elastography remains an uncommon technique at zoos/aquaria and has not yet been applied in marine mammal medicine, likely also due to logistical difficulties of equipment transportation, the requirement for complete patient stillness during the procedure, and the inherent water movement artifacts associated with measurements on aquatic animals.

## Conclusion and Outlook

The diverse imaging modalities discussed here are proven diagnostic tools for assessing mammary gland health in humans and various animal species, including domestic animals and those of zootechnical interest. Despite the clear promise for expanding the fields of marine mammal medicine and comparative reproductive biology, the routine use of these tools for marine mammal health assessments remains challenged largely by equipment accessibility, size of the animals, costs, and other logistical issues. In this context, ultrasonography remains the primary imaging approach in the marine mammal veterinarian’s toolkit, due to its non-invasive nature, real-time evaluation capabilities, and portability, features that are particularly advantageous for examination of aquatic animals. In addition to the need for more flexible, portable and rugged diagnostic tools for field biologists, there is an urgent demand for standardized protocols for evaluating mammary glands in marine mammals, which would facilitate a deeper understanding of the developmental patterns of these organs and help establish reference ranges for their volume and morphological characteristics based on demographic parameters and reproductive stages. The absence of these protocols may originate from limited knowledge transfer between veterinarians and researchers, as imaging modalities are primarily applied in clinical settings and often go unreported in the literature unless a specific case is shared. Better clinician-researcher knowledge transfer would not only support the diagnosis of mammary gland pathologies in marine mammals under human care, but will also support conservation of wild populations. By sharing data more widely, the assessment of mammary glands will also both feed and greatly benefit from the rise of technological developments such as artificial intelligence and machine learning for pathology detection [190–192], paving the way toward more accurate, precise and even automated diagnoses.

Mammary glands are key innovations in the mammalian lineage, more than 300 million years old and probably evolved from apocrine-like glands associated with hair follicles and sebaceous glands in the skin [193, 194]. Although likely arising in the ancestors to modern mammals (synapsids) as secretory organs to help terrestrial eggs resist desiccation, mammary glands evolved an array of specialized structures in support of nourishing young, including placodes, ductal trees, and mammary lines [195]. Moreover, whereas taxa exhibit notable similarities in the various developmental phases of the mammary gland, significant modifications have evolved across mammals especially in early development, resulting in diverse adult morphologies and lactation strategies [8, 195]. A deeper understanding of the selective pressures and changes in developmental processes that have driven mammary gland evolution has been impeded, however, by the limited fossil record for soft tissues and by a lack of knowledge of mammary gland structure, function and development in non-model species (e.g. with diverse reproductive strategies, habitats). This need for integrated understanding calls for broader communication across medical and research disciplines, as well as better communication and sharing of data and data collection approaches. The study of cetacean mammary glands —their morphogenesis, differentiation, and lactation— therefore stands to inform understanding of marine mammal reproduction and health but also, placed in context with other mammal groups, to reveal the evolutionary factors underlying tissue adaptations for novel function and dramatic ecological transitions (e.g. from terrestrial to completely aquatic habitats).

## Data Availability

No datasets were generated or analysed during the current study.
